# Systematic review and meta-analysis of prevalence, trajectories, and clinical outcomes for frailty in COPD

**DOI:** 10.1038/s41533-022-00324-5

**Published:** 2023-01-05

**Authors:** Peter Hanlon, Xuetong Guo, Eveline McGhee, Jim Lewsey, David McAllister, Frances S. Mair

**Affiliations:** School of Health and Wellbeing, University of Glasgow, Glasgow, UK

**Keywords:** Chronic obstructive pulmonary disease, Epidemiology

## Abstract

This systematic review synthesised measurement and prevalence of frailty in COPD and associations between frailty and adverse health outcomes. We searched Medline, Embase and Web of Science (1 January 2001–8 September 2021) for observational studies in adults with COPD assessing frailty prevalence, trajectories, or association with health-related outcomes. We performed narrative synthesis and random-effects meta-analyses. We found 53 eligible studies using 11 different frailty measures. Most common were frailty phenotype (*n* = 32), frailty index (*n* = 5) and Kihon checklist (*n* = 4). Prevalence estimates varied by frailty definitions, setting, and age (2.6–80.9%). Frailty was associated with mortality (5/7 studies), COPD exacerbation (7/11), hospitalisation (3/4), airflow obstruction (11/14), dyspnoea (15/16), COPD severity (10/12), poorer quality of life (3/4) and disability (1/1). In conclusion, frailty is a common among people with COPD and associated with increased risk of adverse outcomes. Proactive identification of frailty may aid risk stratification and identify candidates for targeted intervention.

## Introduction

An increasing number of people worldwide are living with frailty^1^. Frailty describes a state of increased vulnerability to decompensation in response to physiological stress^2,3^. There is growing recognition of the importance of frailty in the management of noncommunicable diseases generally, and chronic respiratory diseases specifically^2,4^. In the context of chronic obstructive pulmonary disease (COPD) (a condition characterised by progressive decline in pulmonary function and periods of exacerbation, both of which may impact on function and independence), it has been argued that frailty may be a valuable concept to understand individual vulnerability to adverse clinical outcomes^5–8^.

While, at a conceptual level, frailty describes a state of increased vulnerability to physiological decompensation, there is no single universally accepted operational measure of frailty. Rather, multiple different measures, drawing on different theoretical models of ageing, have been used to identify frailty within individuals^2^. Populations identified by different frailty definitions only partially overlap^9^. Frailty is also understood to be dynamic and may worsen or improve within individuals over time.

People with COPD are recognised to have a higher prevalence of frailty than the general population^5^. However, this prevalence is likely to differ by different frailty definitions as well as in different clinical settings. A previous systematic review has assessed the prevalence of frailty in people with COPD, however this review did not assess the impact of frailty on clinical outcomes^5^. Moreover, since its publication, a number of further studies focusing specifically on people with COPD, have assessed the prevalence and implications of frailty^10,11^.

This systematic review aims to (i) review the different measures which have been used to quantify frailty in people with COPD; (ii) summarise the prevalence and trajectories of frailty in people with COPD across a range of settings and frailty definitions; and (iii) quantify the relationship between frailty and clinical outcomes in people with COPD.

## Methods

This systematic review of observational studies was carried out according to a pre-specified protocol (PROSPERO CRD42021275574) and is reported according to Preferred Reporting Items for Systematic Reviews and Meta-Analyses guidelines^12,13^.

### Eligibility criteria

Observational studies (cross-sectional or cohort design) meeting the following criteria were included:

- Population: adults aged ≥18 years with COPD;
- Exposure: frailty (any measure);
- Comparison: people with COPD without frailty;
- Outcomes: frailty prevalence (primary outcome), transitions in frailty status, mortality, hospitalisation, healthcare utilisation, quality of life, disability, COPD exacerbation, COPD severity, symptoms;
- Setting: any (including community, outpatients, inpatients, residential care).

There are a wide range of frailty measures described within the published literature. The earliest of these, and the most widely cited, are the frailty phenotype and the frailty index, however many measures have followed. While each of these seeks to identify individuals at greater risk of physiological decompensation and thus adverse health outcomes, measures vary considerably in their theoretical basis, method of assessment, and their quantification (e.g. categorical or continuous, or describing frailty severity). We included studies using any definition of frailty to allow comparison between different measures. Studies were included if they used a validated frailty measure or provided detailed criteria within the manuscript of the criteria used to identify frailty. We excluded studies that assessed frailty based on a single parameter or proxy measure (e.g. grip strength alone). We excluded studies not published in English, conference abstracts, and grey literature. For synthesis, studies were grouped by frailty definition and setting.

### Search strategy

We searched three electronic databases (Medline, Embase and Web of Science Core Collection) using a combination of Medical Subject Headings and keyword searches. The basic search structure was “Frailty” AND “COPD” and was developed by the study authors drawing on previous systematic reviews. Full search terms for Medline are shown in the Supplementary Information and were adapted for the other databases. All search results were exported to Endnote software. Database searches were supplemented by forward citation searches of all eligible studies and hand-searching reference list of relevant papers (included studies and relevant review articles). We did not attempt to contact study authors.

### Study selection

We screened titles and abstract of all records identified through database searching. Full texts of all potentially eligible articles were obtained and screened according to our eligibility criteria. Two reviewers, working independently, completed all stages of screening. Agreement was high between reviewers for the title and abstract screening (kappa statistic 97%). Disagreements over eligibility were resolved by consensus, involving a third reviewer if necessary.

### Data extraction

Two reviewers, working independently, extracted details of study publication (author, year, journal, location), setting (community, outpatient, inpatient, residential care, etc.), population (recruitment method, eligibility criteria, age, sex, socioeconomic status, comorbidities), frailty definition (including adaptations to the original definition), frailty prevalence and the relationship between frailty and any clinical outcomes listed above.

### Quality assessment

The methodological quality of the included studies was assessed using an adaptation of the Newcastle Ottawa Scale (previously adapted for systematic reviews of studies assessing frailty, included in Supplementary Information)^14^. The initial five questions were used for all studies (cross-sectional or longitudinal) with a further six for longitudinal studies.

### Synthesis

We performed a narrative synthesis of frailty prevalence estimates, stratified by study setting and frailty definition. We synthesised studies assessing frailty and clinical outcomes using a combination of narrative synthesis and random effects meta-analysis. Meta-analysis was performed when at least two studies assessed the same outcome, using the same frailty definition, using a similar statistical approach, and when heterogeneity was at an acceptable level (heterogeneity was assessed using the *I*^2^ statistic). Where studies were too heterogeneous, a narrative synthesis was performed and summarised using Harvest plots. Harvest plots summarise heterogenous data using bars to represent individual studies placed on a matrix to indicate where the studies showed a positive, negative, or neutral association with a given outcome (we used *p* < 0.05 to denote statistical significance)^15,16^. Data processing and analysis was done using R (version 3.6.1).

### Reporting summary

Further information on research design is available in the Nature Research Reporting Summary linked to this article.

## Results

**Fig. 1:**
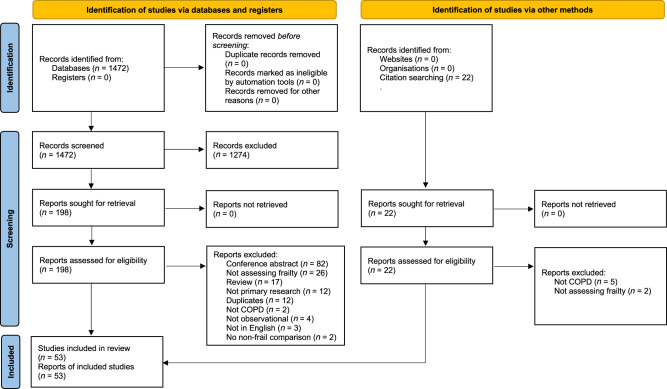
PRISMA diagram of study identification and screening. This figure summarises the stages of identification and screening of eligible studies.

**Fig. 2:**
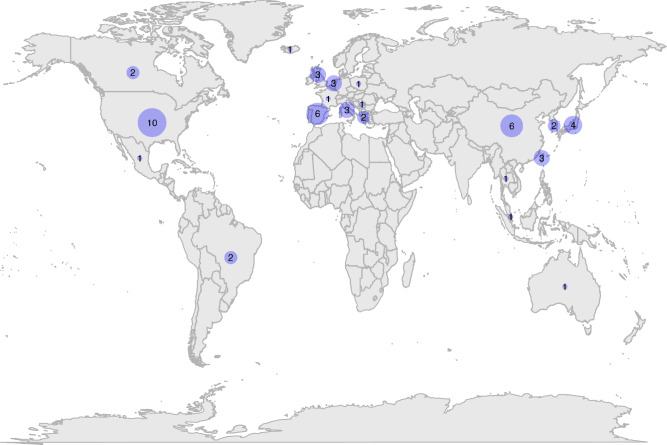
Map showing the location of the included studies. The numbers on the map indicate the number of included studies from each country.

We identified 1402 unique titles and abstracts from electronic database searches, from which we retained 220 for full-text screening and finally identified 53 eligible studies (Fig. 1)^10,11,17–67^. Sample size (with COPD) ranged from 22 to 8074 (median 192, interquartile range (IQR) 103–149). Study characteristics and quality assessment are summarised in Table 1. Mean study age ranged from 50 to 88 (median 73, IQR 68–75). All studies were from high- or upper–middle-income countries (Fig. 2). COPD was identified using a range of definitions, including spirometry-confirmed/GOLD definition, self-reported, and based on electronic medical records. Studies including only spirometry-confirmed COPD tended to be based in respiratory outpatient departments or hospital inpatients and were often single-centre studies. Most community-based studies used self-reported COPD or relied on electronic medical records (Supplementary Information).

**Table 1:** Study characteristics and quality assessment.

Author	Country	Frailty measure	COPD definition	*N* with COPD	Mean age	Setting	Outcomes
Akgun^17^	United States	Frailty phenotype	Electronic health records	182	50	Community	Prevalence
Ambagtsheer^18^	Australia	Frailty index	Electronic health records	98	88	Residential care	Prevalence
Avila-Funes^19^	Mexico	Frailty phenotype	Self report	814	74	Community	Prevalence
Bernabeu-Mora^20^	Spain	Edmonton	GOLD criteria	103	71	Inpatient	Prevalence, hospital readmission, FEV1, dyspnoea
Bernabeu-Mora^21^	Spain	Frailty phenotype	GOLD criteria	119	67	Outpatient	Prevalence, frailty transitions, exacerbation
Blaum^22^	United States	Frailty phenotype	Electronic health records	230	74	Community	Prevalence
Castellana^23^	Italy	Frailty phenotype	Self-report	343	74	Community	Prevalence
Chen^24^	Taiwan	Frailty phenotype	Self-report	312	73	Community	Prevalence
Chen^25^	Taiwan	CFS	Electronic health records	125	77	Outpatient	Prevalence, exacerbation, dyspnoea
Cheong^26^	Singapore	Frailty phenotype	Self-report	239	66	Community	Prevalence
Chin^27^	Canada	CFS	Hospitalised with exacerbation of COPD	50	72	Inpatient	Prevalence
Crow^28^	United States	Frailty phenotype	Self-report	496	71	Community	Prevalence
de Albuquerque^29^	Brazil	Frailty phenotype	Self-report	22	74	Community	Prevalence
Dias^30^	Brazil	FRAIL	GOLD criteria	153	69	Outpatient	Prevalence, exacerbation, FEV1, dyspnoea, CAT
Fragoso^31^	United States	Frailty phenotype	Self-report	262	72	Community	Prevalence, frailty transitions
Fried^32^	United States	Frailty phenotype	Self-report	415	72	Community	Prevalence
Gale^33^	United Kingdom	Frailty index	GOLD criteria	520	66	Community	Prevalence, exacerbation, FEV1, dyspnoea, CAT
Galizia^34^	Italy	Frailty Staging System	Self-report	489	74	Community	Prevalence, mortality, dyspnoea
Gephine^35^	France	Frailty phenotype	GOLD criteria	44	66	Pulmonary rehab	Prevalence, exacerbation, FEV1, dyspnoea, QOL
Gu^36^	China	Frailty index	Clinician diagnosis of acute exacerbation of COPD	154	80	Inpatient	Mortality
Hanlon^37^	United Kingdom	Frailty phenotype	Self-report	8074	58	Community	Prevalence
Hirai^38^	Japan	Kihon	GOLD criteria	201	76	Outpatient	FEV1, dyspnoea, CAT
Ierodiakonou^39^	Greece	FiND	GOLD criteria	257	65	Community	Prevalence, exacerbation, FEV1, dyspnoea, CAT
Kennedy^40^	United States	Frailty phenotype	GOLD criteria	902	68	Outpatient	Prevalence, mortality, hospital admission, QOL
Kim^41^	South Korea	Frailty phenotype	Self-report	83	76	Community	Prevalence
Kusunose^42^	Japan	Kihon	GOLD criteria	79	75	Outpatient	Prevalence, dyspnoea, CAT, QOL
Lahousse^43^	Netherlands	Frailty phenotype	GOLD criteria	172	75	Community	Prevalence
Lahousse^44^	Netherlands	Frailty phenotype	GOLD criteria	172	75	Community	Mortality, FEV1
Lai^45^	Taiwan	Frailty phenotype	Medical records	65	82	Residential care	Prevalence
Lee^46^	China	Frailty phenotype	Self-report	236	74	Community	Frailty transitions
Limpawattana^47^	Thailand	FRAIL	GOLD criteria	121	70	Outpatient	Prevalence
Liotta^48^	Italy	Functional geriatric evaluation	Medical records	218	76	Community	Prevalence
Luo^10^	China	Frailty phenotype	GOLD criteria	309	86	Outpatient	Prevalence, mortality, exacerbation, hospital admission, FEV1, dyspnoea, CAT
Ma^49^	China	Frailty index	Self-report	205	72	Community	Prevalence
Maddocks^50^	United Kingdom	Frailty phenotype	GOLD criteria	816	70	Pulmonary rehab	Prevalence, frailty transitions, exacerbation
Medina-Mirapeix^51^	Spain	Edmonton	GOLD criteria	103	71	Inpatient	Prevalence, disability
Medina-Mirapeix^52^	Spain	Frailty phenotype	GOLD criteria	137	67	Outpatient	Prevalence, exacerbation, FEV1, dyspnoea, CAT
Motokawa^53^	Japan	Kihon	Self-report	6	74	Community	Prevalence
Nagorni-Obradovic^54^	Serbia	Frailty phenotype	Self-report	653	59	Community	Prevalence
Oishi^55^	Japan	Kihon	American Thoracic Society guidelines	128	73	Outpatient	Prevalence, dyspnoea
Park^56^	United States	Tilburg	Self-report	211	71	Community	Prevalence, dyspnoea
Park^57^	South Korea	Tilburg	Self-report	417	65	Community	Prevalence, FEV1
Pollack^58^	United States	Frailty phenotype	Self-report	537	73	Community	Prevalence
Scarlata^59^	Italy	Frailty index	GOLD criteria	150	73	Outpatient	Prevalence, mortality, exacerbation, FEV1, dyspnoea, CAT
Serra-Prat^60^	Spain	Frailty phenotype	Self-report	44	80	Community	Prevalence
ter Beek^61^	Netherlands	Frailty phenotype	GOLD criteria	57	61	Pulmonary rehab	Prevalence
Uchmanowicz^62^	Poland	Tilburg	American Thoracic Society guidelines	102	63	Outpatient	Prevalence
Valenza^63^	Spain	Frailty phenotype	American Thoracic Society guidelines	212	73	Inpatient	Prevalence
Veronese^64^	Iceland	Frailty phenotype	Self-report	368	76	Community	Prevalence
Warwick^65^	Canada	CFS	Hospitalised with exacerbation of COPD	390	68	Inpatient (ITU)	Prevalence, mortality
Xue^66^	China	Frailty phenotype	Self-report	23	79	Outpatient	Prevalence
Yee^11^	United States	Frailty phenotype	GOLD criteria	280	68	Outpatient	Prevalence, mortality, exacerbation, hospital admission, FEV1, QOL
Zhang^67^	China	Frailty phenotype	Self-report	28	75	Outpatient	Prevalence

*GOLD* Global Obstructive Lung Disease, *FEV*1 Forced expiratory volume in 1 second, *CAT* COPD Assessment Test, *QOL* quality of life.

### Quality assessment

**Fig. 3:**
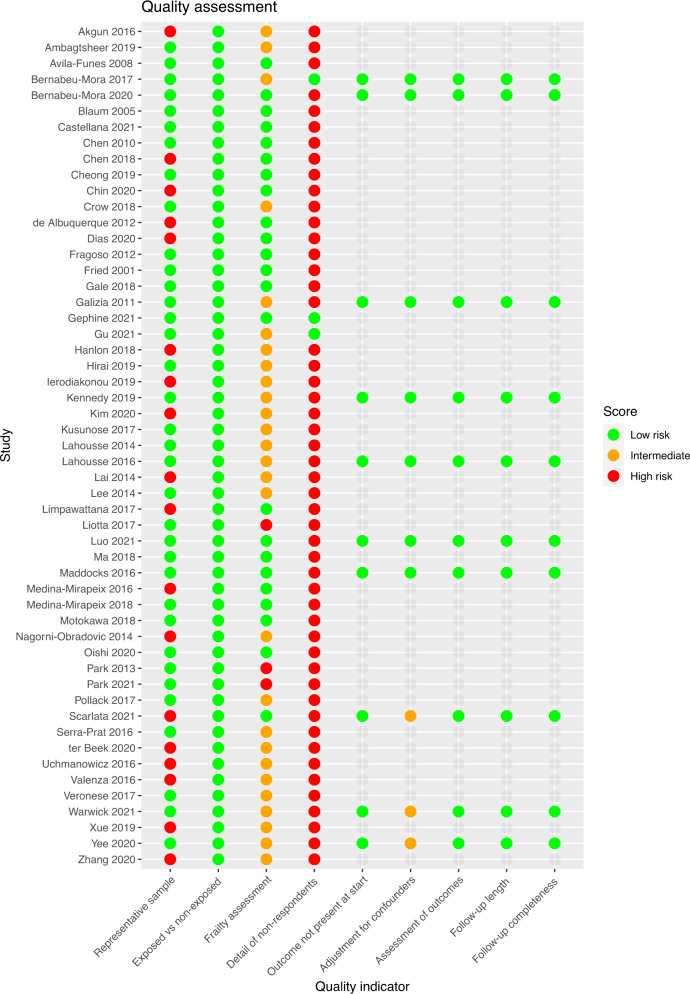
Quality assessment of the included studies. Coloured dots indicate the quality assessment for each domain based on the Newcastle Ottawa quality assessment scale (full details in given in the supplementary material).

The results of the quality assessment for each study are shown in Fig. 3, with additional detail in Supplementary Information. While many studies were judged somewhat representative (e.g. through consecutive non-selective recruitment of a clinical population, or use of a representative community-based sample), many of these studies still had exclusion criteria with the potential to influence the prevalence of frailty in these studies. For example, many studies excluded people with cognitive impairment (sometimes not specifying the criteria or cut-off used to judge this), people with ‘impaired mobility’ or with some specific long-term conditions (e.g. neurological conditions such as stroke or Parkinson’s disease). While these resulted in few overall exclusions and may be justified given the demands of the study (e.g. in terms of physical assessments), given the associations between these features and frailty it is likely that these criteria would selectively exclude people living with frailty from these studies. This, in turn, may influence prevalence estimates.

Many studies adapted existing frailty criteria based on their available data. However, frailty measures used were well described in most studies. Very few studies provided any detail of differences between participants with and without missing data or between non-responders and those with complete follow-up.

In the longitudinal studies assessed, all adjusted for age and sex in analyses and all but one adjusted for other relevant covariates (e.g. COPD severity). Length and completeness of follow-up was judged adequate for outcome assessment (Supplementary Table 2).

### Frailty measurement

The included studies used a total of 11 different frailty measures. The most common was the frailty phenotype^32^ (32 studies) followed by the frailty index^68,69^ (5 studies), Kihon checklist^70^ (4 studies), Clinical Frailty Scale^71^ (3 studies), Tilburg frailty indicator^72^ (3 studies), FRAIL scale^73^ (2 studies), Edmonton frailty indicator^74^ (2 studies), FiND^75^ (1 study), Study for Osteoporotic Fractures frailty score^76^ (1 study), Frailty staging system^77^ (1 study) and Functional Geriatric Evaluation^78^ (1 study). One study assessed used three different measures^38^. These definitions are summarised in Table 2. While the frailty phenotype was the most commonly used measure, 27 of the 32 studies using this measure made some adaptation to the original frailty phenotype criteria.

**Table 2:** Frailty measures used in the included studies.

Frailty measure	Components	Range and categorisation	Number of included studies
Frailty phenotype^32^	5 components (unintentional weight loss, exhaustion, low grip strength, slow walking pace, low physical activity)	1–2 criteria: pre-frail ≥3 criteria: frail	32
Frailty index^68,69^	Count of health-related deficits (≥30, type and number of chosen deficits may vary between studies). Total present divided by number of possible deficits	Range 0–1 Sometimes categorised (threshold for frailty varies (e.g. 0.2, 0.24)	5
Kihon checklist^70^	Self-administered checklist (components: activities of daily living, exercise, falling, nutrition, oral health, cognition, depression)	Unweighted sum of components. Range 0–25. Pre-frail (4–7), Frail (≥8)	4
Clinical frailty scale^71^	Clinical tool based on functional status	Ranges 1 (very fit) to 9 (terminally ill). Some dichotomise as ≥5 = frail	3
Tilburg frailty indicator^72^	15 questions across 3 domains (physical, psychological and social) Responses combined into unweighted sum	Range 0–15 ≥5 indicates frailty	3
FRAIL scale^73^	5 components (weight loss, fatigue, weakness, ambulation, illness/comorbidity)	1–2 criteria: pre-frail ≥3 criteria: frail	2
Edmonton frailty scale^74^	9 components: cognition, general health, functional independence, social support, medication, nutrition, mood, continence and functional performance	Score 0–17. Mild (7–8), moderate (9–10) and severe frailty (≥11)	2
FiND^75^	Self-administered frailty screening tool. 5 items: difficulty walking 400 m, difficulty climbing stairs, weight loss, exhaustion and low physical activity	Classed as disability (difficult walking or climbing stairs), frailty (no difficulty with walking or stairs but other deficits present) or robust (no deficits)	1
Study of Osteoporotic Fracture frailty indicator^76^	3 components (weight loss, chair stand, exhaustion)	1 component: pre-frail 2–3 components: frail	1
Frailty staging system^77^	7 components (disability, mobility, cognition, vision, hearing, continence, social support)	Range 0–7. Mild (1) moderate (2–3) or severe frailty (≥4)	1
Functional geriatric assessment^78^	Multidimensional evaluation (physical, mental and functional status, socio/economic resources, environment)	Scored from −108 to 101 Robust (>50), frail (≤50, ≥10) and very frail (<10)	1

Table adapted from Hanlon et al.^14^.

### Frailty prevalence

**Fig. 4:**
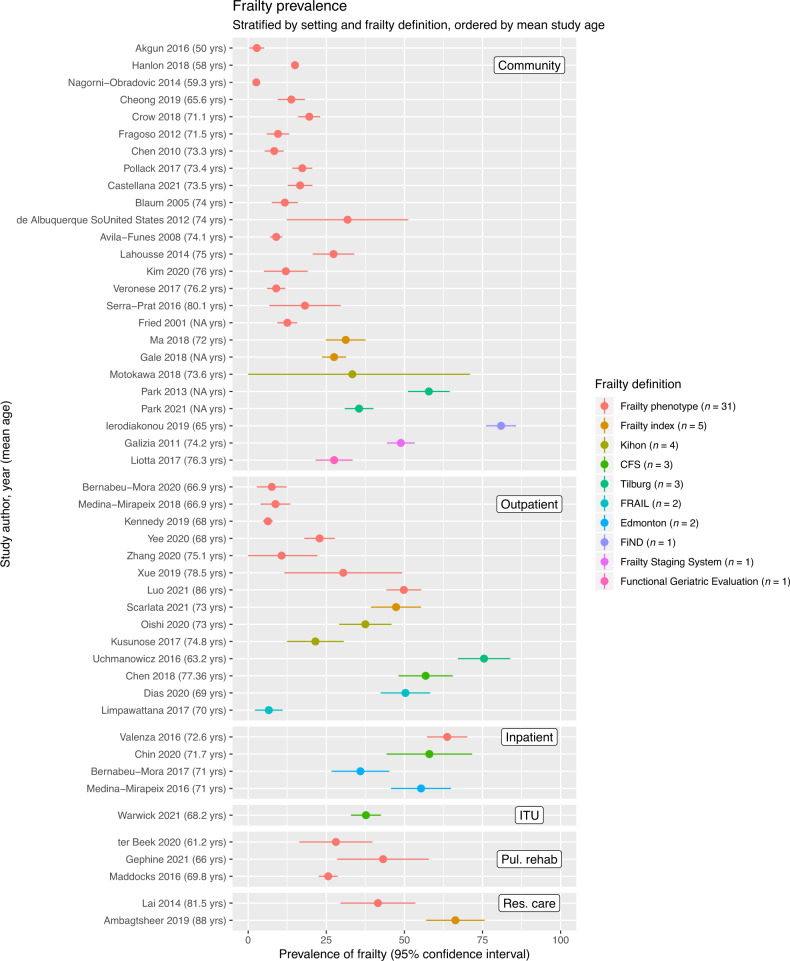
Plot showing the prevalence of frailty in each of the included studies. Studies are stratified by setting (community, outpatient, inpatient, intensive care (ITU), pulmonary rehabilitation and residential care). Within strata, studies are ordered by frailty measure (colour) and mean age (descending order on y-axis). Points indicate the prevalence estimate, lines indicate 95% confidence intervals.

The prevalence of frailty was assessed in 47 studies. These are summarised in Fig. 4, stratified by study setting and ordered by frailty definition and mean age in the included studies. Estimates of prevalence were highly heterogeneous, varying by setting and between frailty definitions. Prevalence ranged from 2.6 to 80.9% in 25 community-based studies and from 6.3 to 75.5% in 14 studies in outpatient settings. Prevalence varied by frailty measure (e.g. generally lower in studies using frailty phenotype). Higher mean age of the sample appeared to be associated with a higher frailty prevalence, while some younger populations (mean age 50–60 years) had considerably lower prevalence estimates, for example among community-based studies using the frailty phenotype (Fig. 4). However, even among community-based studies using the same measure there was heterogeneity in prevalence estimates among samples of a similar age, suggesting that additional factors (such as population demographics or adaptations in the way frailty deficits were specified) also influenced the prevalence of frailty. In other settings, prevalence ranged from 35.9 to 63.7% in hospital inpatients (4 studies), from 41.5 to 66.3% in residential care settings (2 studies) and 25.6 to 43.2% in populations recruited at the commencement of pulmonary rehabilitation programmes (3 studies). Some of the highest frailty estimates were from studies in which the original frailty definition was adapted based on available data and the cut-point for identifying frailty was selected by the study authors (rather than being based on externally validated criteria). This may explain some of the notably high prevalence estimates and limits the generalisability of these studies.

### Within-person frailty trajectories

Four studies assessed longitudinal changes in frailty over time. These varied in their setting, aim, and design. Two studies focused exclusively on people with COPD. Bernabeu-Mora et al. (*n* = 119) found that higher baseline muscle strength and lower exacerbation frequency were associated with improvement, while higher baseline dyspnoea was associated with worsening frailty status. Maddocks et al (*n* = 816) found that people living with frailty were more likely not to complete pulmonary rehabilitation due to exacerbations or hospital admissions; however, of those who did complete, 71/115 (61.3%) no longer met the criteria for frailty (74 pre-frail, 7 robust). People with baseline frailty also experienced greater improvements in dyspnoea, physical activity and health status following pulmonary rehabilitation than people who were not living with frailty at baseline.

Of the two studies of frailty trajectories among general populations (not limited to people with COPD), COPD was associated with worsening frailty status among women who were robust at baseline (adjusted odds ratio (OR) 2.66, 95% confidence interval (CI) 1.10–6.44); however, they did not find the same association in men (adjusted OR 0.61, 95% CI 0.34–1.11). COPD defined by obstructive spirometry was associated with worsening frailty status compared to people with no airway obstruction over 3 years follow-up (adjusted OR 1.58; 95% CI 1.17–2.13). Baseline frailty status was also associated with the development of respiratory impairment (defined as obstructive or restrictive pattern of spirometry) among people with no respiratory impairment at baseline (adjusted OR 1.42; 95% CI 1.11–1.82).

### Frailty and clinical outcomes

**Fig. 5:**
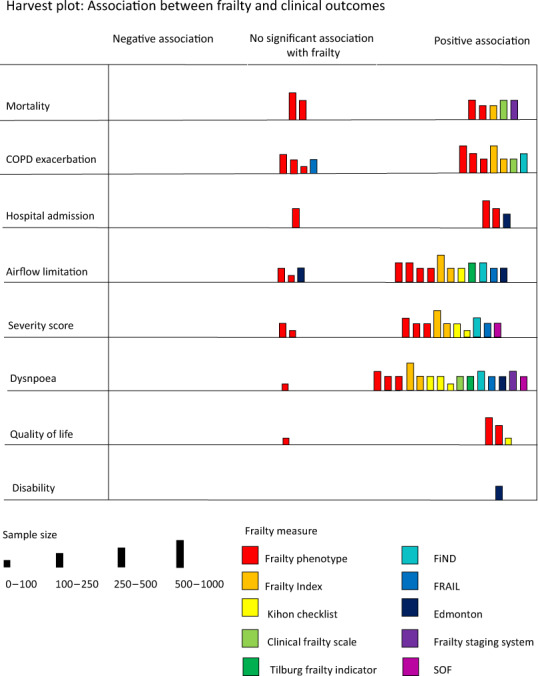
Harvest plot showing the relationship between frailty and clinical outcomes in the included studies. Each bar represents a single study. Colour is used to indicate which frailty measure is used. Height of the bar indicates study sample size. The position of the bar on the matrix indicates the relationship between frailty and the outcome in question (positive association between frailty and the outcome, no significant association, or negative association between frailty and the outcome [i.e. frailty is protective]).

Studies assessing the relationship between frailty and clinical outcomes (either cross-sectionally or prospectively) are summarised in Fig. 5. Detail of each of the results displayed in Fig. 5 (including analysis and effect sizes) is summarised in the Supplementary Information. These are explored in detail below.

#### Mortality

Eight studies assessed the relationship between frailty and all-cause mortality in people with COPD.

**Fig. 6:**
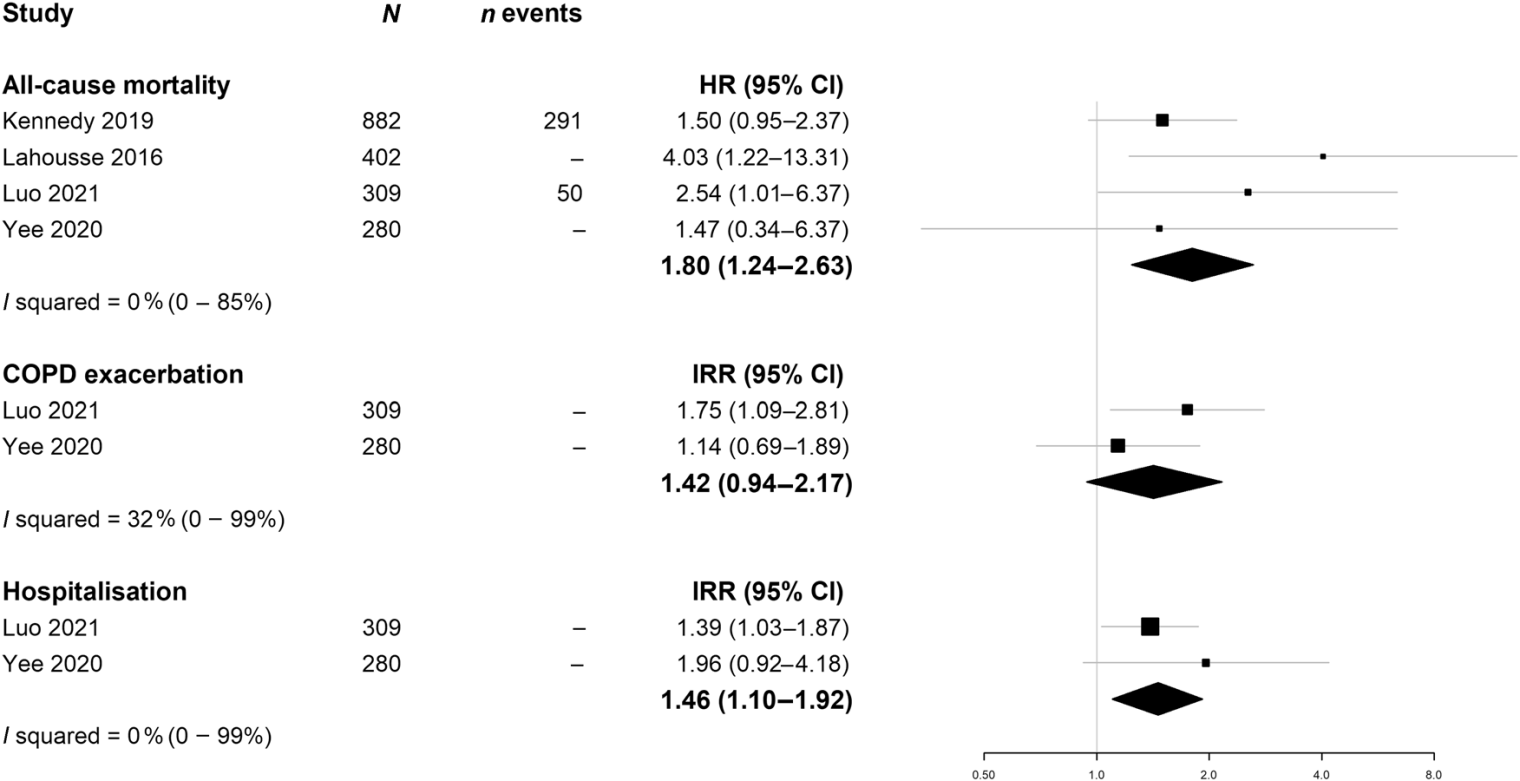
Meta-analyses of random-effects meta-analysis of the relationship between frailty phenotype (frailty phenotype definition) and mortality, COPD exacerbation and hospitalisation. Points indicate hazard ratios (HR) or incidence rate ratios (IRR), respectively. Whiskers show 95% confidence intervals (CI).

Four of these were outpatient- or community-based studies that used the frailty phenotype definition. The pooled hazard ratio (HR) for mortality associated with frail compared to robust participants was 1.80; 95% CI 1.24–2.62 (Fig. 6). Statistical heterogeneity was low (*I*^2^ = 0%) despite variation in study location (2 USA, 1 China, 1 Netherlands), adaptation of the frailty phenotype criteria and covariates included in the adjusted models. However, given the small number of studies included, this assessment of heterogeneity may be imprecise (95% CI 0–85%). One other community study (*n* = 489) using the Frailty Staging system also showed an association between frailty and mortality. In an outpatient-based study using the frailty index (*n* = 150), the association between frailty and mortality was not statistically significant (HR 2.1; 95% CI 0.7–5.8).

Two studies assessed inpatient mortality. One used the Clinical Frailty Scale and assessed survival to discharge from intensive care (*n* = 390). The other used a Frailty Index based on laboratory measures and compared people surviving to hospital discharge (*n* = 77) with people who did not (*n* = 77) using propensity score matching. In both studies, a higher degree of frailty was associated with higher mortality.

#### Hospitalisation

Three outpatient-based studies assessed the relationship between the frailty phenotype and all-cause hospital admission. Hospitalisation rates among people living with frailty and COPD were high (2 studies reported mean 0.49 and 0.47 exacerbations per person per year, respectively, in people living with frailty compared to 0.20 and 0.21, respectively, in robust participants with COPD). In the two studies assessing the rate of exacerbations, the pooled incident rate ratio (IRR) was 1.46, 95% CI 1.10–1.92. In the remaining study, the adjusted HR was 1.8, 95% CI 1.1–2.9. A fourth study assessed the relationship between frailty (Edmonton frailty indicator) and readmission within 90 days of COPD exacerbation. Severe frailty was strongly associated with readmissions (45% compared to 18% of robust participants, adjusted OR 5.19, 95% CI 1.26–21.50). Taken together, there is consistent evidence that people living with frailty and COPD experience higher rates of hospital admission than robust individuals with COPD.

#### COPD exacerbation

Eleven studies reported the association between frailty status and COPD exacerbations. Only two of these were prospective studies, both of which used the frailty phenotype definition. On meta-analysing these two studies, incidence of COPD exacerbations was 42% higher in those who were frail compared to those who were not (IRR 1.42, 95% CI 0.94–2.17, see Fig. 6). The remaining 9 studies assessed the unadjusted association between baseline frailty status and exacerbation frequency in the period prior to baseline (typically number of exacerbations, or frequent exacerbations defined as ≥2, in the past year). Six of these nine studies reported an association between frailty and exacerbation frequency.

#### COPD severity

Fourteen studies assessed the cross-sectional relationship between frailty and either forced expiratory volume in 1 s (FEV1), FEV1 as a percentage of predicted or severity categories based on FEV1. Frailty was associated with a greater degree of airflow limitation in 11/14 of these studies. Seven studies reported frailty prevalence stratified by degree of airflow limitation, showing higher frailty prevalence in participants with more severe airflow limitation (Supplementary Information).

Eight studies, comprising seven different frailty measures, assessed the relationship between frailty and CAT score. All of these reported statistically significant (unadjusted) baseline associations between frailty and higher CAT scores.

#### Dyspnoea

Of the 16 studies comparing baseline dyspnoea in people with and without frailty, 13 used the modified MRC dyspnoea scale, of which 12 showed greater dyspnoea scores in people living with frailty. Three remaining studies also reported cross-sectional associations between frailty and self-reported dyspnoea. As mentioned above, baseline dyspnoea was also associated with worsening frailty status over 2 years follow-up in the only longitudinal study to assess this outcome.

#### Quality of life

Four studies assessed the cross-sectional relationship between frailty and quality of life. Three of these, all of which used the short-form 36 quality of life assessment, reported lower quality of life scores in participants with baseline frailty (2 frailty phenotype, 1 Kihon checklist). The remaining study used was small (*n* = 55) and found no evidence of association between the frailty phenotype and quality of life according to the CCQ.

#### Disability

A single study assessed trajectories of activities of daily living limitation following hospital admission with COPD exacerbation. Frailty, measured using the Edmonton frailty indicator at the point of admission, was statistically significantly associated with functional decline measured 12 weeks after discharge (adjusted OR 3.97; 95% CI 1.13–13.92).

## Discussion

This systematic review summarises the existing literature on the prevalence, trajectories and clinical implications of frailty in people with COPD. The prevalence of frailty varies by setting, age and by method used to identify frailty, ranging between 2.6 and 80.9%. Nonetheless, our findings show that frailty is common among people with COPD. Older populations had a higher prevalence of frailty, as expected; however, frailty remained common in populations of people with COPD including those aged under 65 years. In inpatient settings, the prevalence of frailty was notably higher (between 36 and 64% using a variety of measures), as it was in people living in residential care and in pulmonary rehabilitation.

Our findings demonstrate that people living with COPD and frailty may experience a high frequency of exacerbations, tend to have more severe airflow limitation, higher prevalence of dyspnoea and are at greater risk of readmission following hospital discharge and of mortality. Frailty in people with COPD varies over time.

A previous systematic review, including 27 studies, assessed the prevalence of frailty in COPD but did not synthesise the relationship between frailty and clinical outcomes in COPD^5^. In this previous review, 13 studies used the frailty phenotype, with a pooled mean prevalence of 19%, 95% CI 14–24%. Heterogeneity of these estimates was high. This previous study also reported that people with COPD had a twofold higher odds of frailty prevalence than people without COPD (odds ratio 1.97, 95% CI 1.53–2.53). Our study builds on these findings by stratifying by setting and also includes several more recent studies that specifically focussed on frailty in COPD (rather than as a subset of a general population). Unlike the previous review, we did not meta-analyse prevalence estimates due to considerable variation in study inclusion criteria (e.g. the exclusion of people with cognitive impairment or mobility difficulty), age range and adaptations of the frailty phenotype criteria. These sources of heterogeneity limit the interpretability of a single pooled prevalence estimate.

The high prevalence of frailty in people with COPD likely represents complex inter-relationships between features of both frailty and COPD^79–81^. COPD gives rise to a range of extrapulmonary manifestations, such as sarcopenia and fatigue^80,81^, which may contribute to the manifestation of frailty. Depending on the frailty definition, these factors alone may be sufficient for an individual to be classified as frail (i.e. COPD may cause frailty, in this context)^82,83^. Conversely, the development of COPD itself involves a complex interplay between environmental exposures and genetic susceptibility^84^. Accelerated lung ageing has been proposed as one mechanism underlying this process^85^. Some have argued that the development of COPD should be best understood as an interplay between abnormalities in organ development and maintenance (susceptibility) and the cumulative effect of tissue injury and ageing^84,86^. This is similar paradigm to the cumulative deficit model of frailty that is operationalised in the frailty index^68,69,83^. Under this framework, the development of COPD is a manifestation of the same processes underlying frailty, rather than a cause of frailty itself. In summary, depending on the theoretical model used to define frailty, COPD may be understood as both a cause and a consequence of frailty.

While frailty may result from the cumulative effect of acquired deficits, it may improve as well as worsen over time^87^. This review highlights that COPD may be a risk factor for worsening frailty; however, it also demonstrates that frailty in COPD may be responsive to targeted intervention, specifically pulmonary rehabilitation^50^. One study in this review also suggested that factors (such as COPD) associated with frailty transitions may vary by gender; however, this study tested a large number of covariates and was also limited the range of comorbidities that were measured; therefore, multiple testing resulting in false positives or residual confounding remain possible alternative explanations^42^. Sources of variation in frailty trajectories, including underlying long-term conditions and demographic factors such as gender are an important area of future study. The improvement in frailty status with pulmonary rehabilitation was assessed in only one included study. However, these findings are consistent with emerging evidence on interventions targeting frailty in general populations. A recent meta-analysis of 46 interventions showed that interventions incorporating physical activity or nutritional supplementation could improve frailty status in some individuals^88^.

The wide range of frailty definitions available, and lack of consensus over an optimal definition, is well described within the frailty literature. These measures vary considerably in their method of assessment and in their theoretical basis. Our findings highlight that these measures may differ considerably in their prevalence estimates, and therefore choice of measure used to identify frailty is likely to directly influence identification whether used for research or for clinical purposes (e.g. risk stratification). Despite this, the relationship with COPD severity and with adverse outcomes was broadly shared across frailty definitions. Therefore, while populations identified as living with frailty may differ, those measures available do each appear to identify higher risk of adverse outcomes. Choice of measure used to identify frailty should therefore be informed by several factors including practicality of administration and the theoretical basis for identifying frailty. For example, a study seeking to understand how risk factors or physiological parameters affect frailty development may favour a tightly defined physical measure of frailty (e.g. frailty phenotype), whereas studies seeking to assess patients most in need to a multidisciplinary clinical intervention may favour a broader definition incorporating additional domains, such as cognition, nutrition and social vulnerability. In either case, our findings suggest that applying a well-validated frailty measure using explicit cut-offs based on external studies is likely to provide a more reliable frailty assessment.

The high prevalence of frailty among people admitted to hospital with COPD, coupled with the higher rates of hospital readmission, indicate that identifying frailty within respiratory inpatient services may offer opportunities for targeted interventions. However, it is likely that a high proportion of patients would meet the criteria for frailty. It is likely that appropriate clinical response will vary between individuals identified as frail. Individualisation of care, as well as holistic multidisciplinary care, are central tenets of frailty care. There is considerable overlap between these principles and the multidisciplinary care advocated for people with COPD. However, given the high prevalence of frailty, implementing this in practice requires considerable resource and coordination of multiple professionals.

Community prevalence of frailty was lower than among inpatients but still common and associated with adverse events, so proactive identification of frailty may aid risk stratification and identification of individuals for whom limited community resources may be targeted. Ideally, such effort should be integrated into existing systems for the monitoring and management of COPD, to minimise the additional burden on both patients and professionals. Responses to frailty in this context may include advance care planning as well as efforts to maximise function and identify potentially reversible aspects of frailty. Understanding factors that influence trajectories of frailty is an important area for future research, to inform the design and delivery of interventions. Also vital to these efforts would be exploring factors that may facilitate or act as barriers to participation in interventions designed to target frailty.

Strengths of this review include a comprehensive search strategy supplemented by hand-searching reference lists and forward citation searching, followed by duplicate screening and data extraction. However, this review is limited by the exclusion of articles not published in English and of grey literature. As with any systematic review, there is a risk of publication bias. As the number of studies included in meta-analyses was small, assessment of funnel plots to detect publication bias may be unreliable. The included studies themselves are all observational, meaning the observed relationships between frailty and clinical outcomes, or between COPD and frailty trajectories, cannot be assumed to be causal. Many studies defined COPD using either self-report or by coded diagnosis, and there is therefore a risk of some misclassification in COPD in these studies. Spirometry confirmed COPD was mostly limited to studies conducted in respiratory outpatient departments or hospital inpatient settings. While these studies used validated, and therefore more reliable, definitions of COPD most were single centre and the frailty prevalence estimates may not be as generalisable to other settings. Most studies had small sample sizes. Studies from lower–middle-income counties were lacking, limiting the generalisability of prevalence estimates. Furthermore, most of the studies assessing airflow limitation, COPD severity and exacerbations were cross-sectional, further limiting inferences about the consequences of frailty in people with COPD. Most of the included studies contained detailed description of the population, and prospective studies adjusted for relevant potential confounders. However, description of non-responders and loss to follow-up was often limited. Furthermore, the potential confounders included in models assessing prospective outcomes (mortality, hospitalisations and exacerbations) varied widely between studies. Most notably, several did not adjust for severity of airflow limitation. Finally, the included studies were highly heterogenous in terms of inclusion criteria, frailty definitions, setting, and diagnostic criteria for COPD. Therefore, even among studies using the same model of frailty, differences in study populations and adaptation of frailty definition limited the synthesis of prevalence estimates.

Frailty is common in people with COPD and associated with disease severity, symptom burden and with a range of adverse health outcomes. Frailty varies over time and may be responsive to interventions. Proactive identification of frailty may aid risk stratification and identification of individuals for whom interventions may be targeted.

## Supplementary information

Supplementary Material

Reporting Summary

Supplemental Material File #1

## Data Availability

Extracted data reported in the manuscript are contained within the Supplementary Information.
