# Supplementary material for: Systematic review and meta-analysis of prevalence, trajectories, and clinical outcomes for frailty in COPD
Source: NPJ Prim Care Respir Med. 2023 Jan 5;33:1. doi: 10.1038/s41533-022-00324-5 (PMC9816100; doi:10.1038/s41533-022-00324-5)
Supplement: Supplementary file 1 — Supplementary Material [file 41533_2022_324_MOESM1_ESM.pdf]

Supplementary appendix

Table of Contents

Search Strategy 2
The Newcastle-Ottawa Scale adaptation 3
1 – Representativeness of the exposed (i.e. frail) cohort 3
2 – Selection of the non-exposed (i.e. non-frail) cohort 3
3 – Ascertainment of exposure 3
4 – Non-respondents 3
5 – Demonstration that outcome of interest was not present at the start of the study 3
Comparability: 3
1 – Comparability of the cohorts on the basis of the design or analysis being controlled for confounders 3
Outcomes: 4
1 – Assessment of outcomes 4
2 – Follow-up long enough for outcomes to occur 4
3 – Adequacy of follow-up of cohorts 4
Supplementary figure 1 - Prevalence estimates (spirometry-defined COPD) 5
Supplementary figure 2 - Frailty prevalence stratified by COPD severity 6
Supplementary table 1 - association between frailty and adverse health outcomes 7
Funnel plots 15
Supplementary figure 3 - Mortality meta-analysis funnel plot 15
Supplementary figure 4 - Hospitalisation meta-analysis funnel plot 15
Supplementary figure 5 - COPD exacerbation meta-analysis funnel plot 16
Supplementary table 2 - Quality assessment individual study assessments 17

Search Strategy

Medline Search Strategy (adapted for other databases):

1 exp Lung Diseases, Obstructive (MeSH)
2 exp Pulmonary Disease, Chronic Obstructive (MeSH)
3 emphysema$.mp.
4 (chronic$ adj3 bronchiti$).mp.
5 (obstruct$ adj3 (pulmonary or lung$ or airway$ or airflow$ or bronch$ or respirat$)).mp.
6 (COPD or COAD or COBD).mp.
7 1 or 2 or 3 or 4 or 5 or 6

8 exp Frailty/ (MeSH)
9 exp Frail Elderly/ (MeSH)
10 frail$.tw.
11 8 or 9 or 10

12 7 and 11

Search conducted from inception to September 2021 in all databases

MeSH: Medical Subject Heading
$: Truncation tool
Adj3: adjacent (within 3 words)

The Newcastle-Ottawa Scale adaptation

Adaptation for studies assessing the prevalence and impact of frailty in COPD

1 – Representativeness of the exposed (i.e. frail) cohort

a) Truly representative (one star)

b) Somewhat representative (one star)

c) Selected group

d) No description of the derivation of the cohort

2 – Selection of the non-exposed (i.e. non-frail) cohort

a) Drawn from the same community as the exposed cohort (one star)

b) Drawn from a different source

c) No description of the derivation of the non-exposed cohort

3 – Ascertainment of exposure

a) Validated measurement tool for frailty (two stars)

b) Non-validated measurement tool, but the tool is available or described (one star)

c) No description of measurement tool

4 – Non-respondents

a) Comparability between respondents and non-respondents' characteristics is established, and the response rate is satisfactory (one star)
b) The response rate is unsatisfactory, or the comparability between respondents and non-respondents is unsatisfactory

c) No description of the response rate of the characteristics of the responders and non-responders

5 – Demonstration that outcome of interest was not present at the start of the study

a) Yes (one star)

b) No

Comparability:

1 – Comparability of the cohorts on the basis of the design or analysis being controlled for confounders

a) The study controls for age and sex (one star)

b) The study controls for other factors (one star)

c) Cohorts are not comparable on the basis of the design or analysis controlled for confounders

Outcomes:

1 – Assessment of outcomes

a) Independent assessment (one star)

b) Record linkage (one star)

c) Self-report

d) No description

e) Other

2 – Follow-up long enough for outcomes to occur

a) Yes (one star)

b) No

3 – Adequacy of follow-up of cohorts

a) Complete follow-up: all subjects accounted for (one star)

b) Subjects lost to follow-up unlikely to introduce bias – number lost less than or equal to 20% or description of those lost suggested no different from those followed (one star)

c) Follow-up rate less than 80% and no description of those lost

d) No statement

Supplementary figure 1 - Prevalence estimates (spirometry-defined COPD)

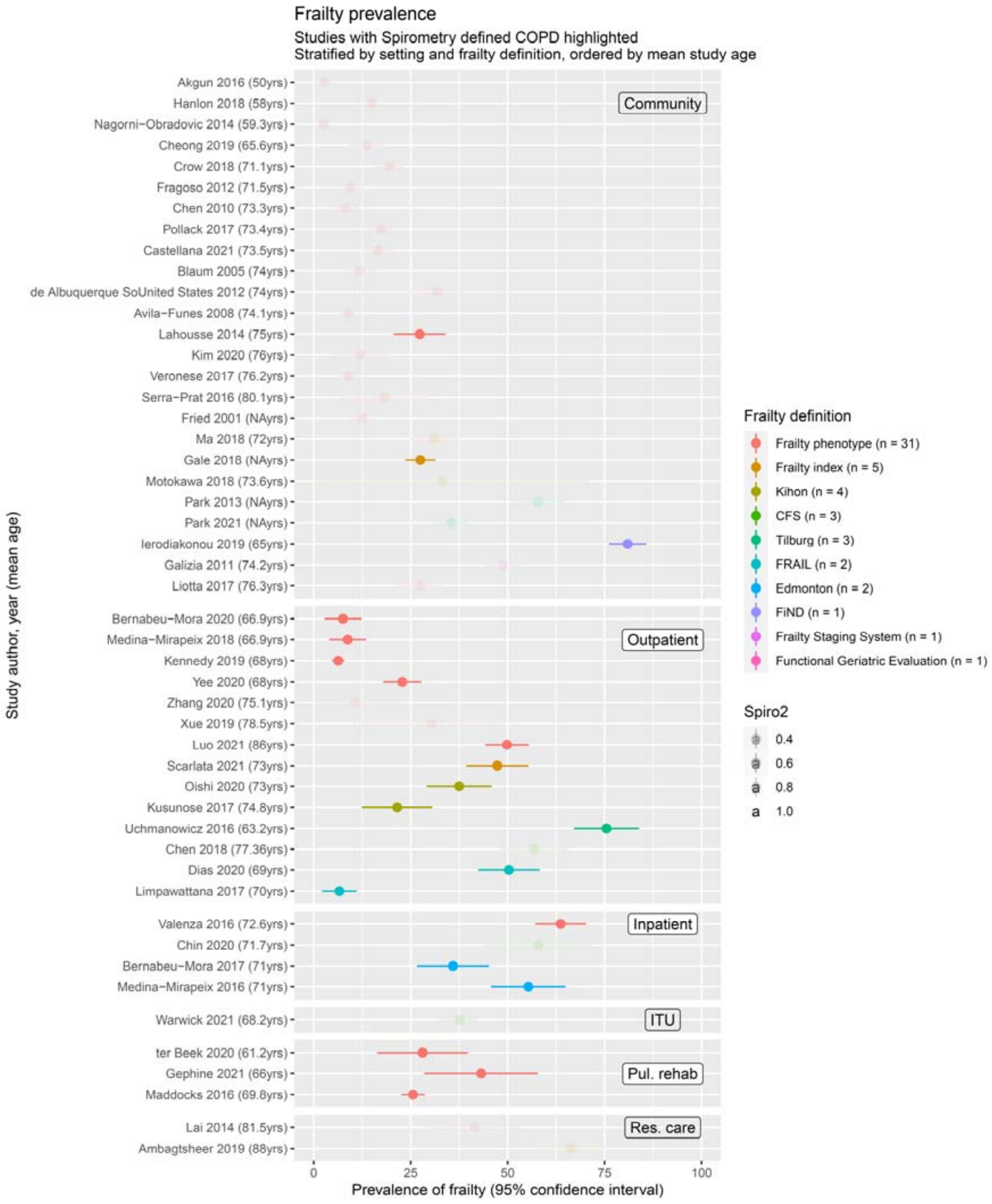

Supplementary figure 2 - Frailty prevalence stratified by COPD severity

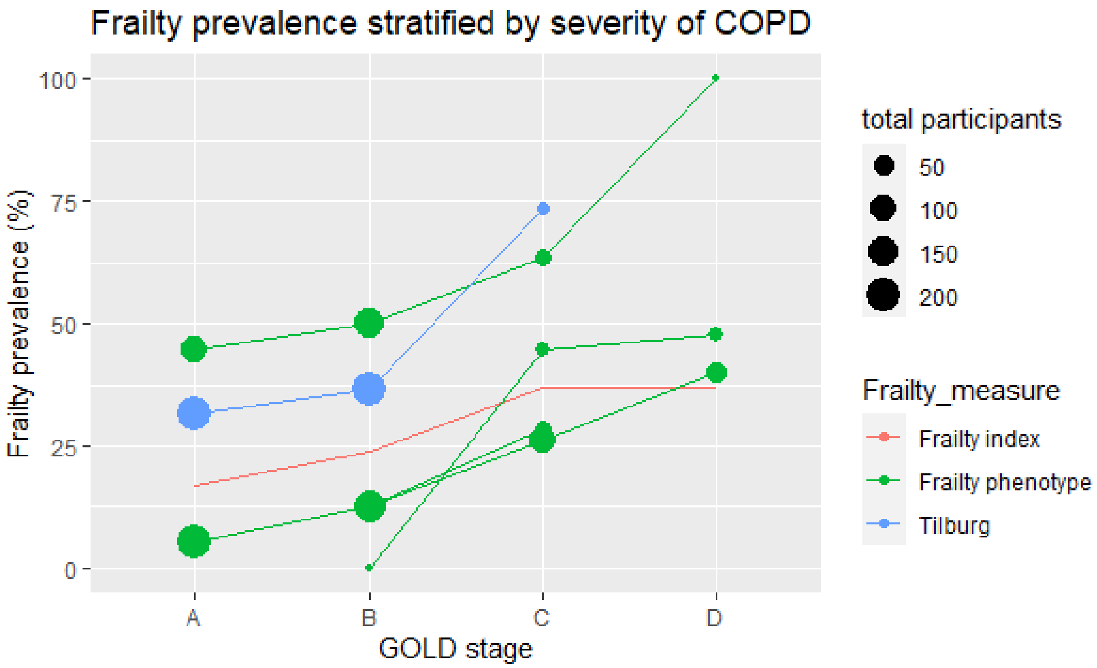

Supplementary table 1 - association between frailty and adverse health outcomes

Author, Year	Frailty measure	Outcome	Total N (n events, if available))	Analysis	Effect size	Harvest plot judgement
Mortality						
Galizia 2011	Frailty staging system	mortality	489	cox PH models adjusted for age, sex, BMI, heart rate, pulse pressure, effort dyspnea, Charlson comorbidity index, drug number, β-agonists, steroids, current smoking, former smoking, MMSE, GDS, BADL (≥1 lost) and social support	HR 1.80 (1.28-2.53)	positive association
Kennedy 2019	Frailty phenotype (adapted)	mortality	902 (291)	coxph model adjusted for components of the ADO index (age, modified Medical Research Council [mMRC] scale, and baseline forced expiratory volume in 1 second [FEV1]) sex, and lung volume reduction surgery	HR 1.5 (0.96-2.4)	neutral association
Lahousse 2016	Frailty phenotype	mortality	402	CoxPH model adjusted for age, sex, BMI, smoking status, pack-years, and the comorbidity count. Models were adjusted for covariates that changed the point estimate by more than 5%	HR 4.03 (1.22-13.30)	positive association
Yee 2020	Frailty phenotype (adapted)	mortality	280 (21)	CoxPH model adjusted for age, sex, Charlson comorbidity index ≥1, supplemental home oxygen use, COPD hospitalizations in the prior year, FEV1%predicted, and mMRC score	HR 1.47 (0.34-6.38)	neutral association
Luo 2021	Frailty phenotype	mortality	309	CoxPH model adjusted for age, gender, CCI, medication, GOLD severity, moderate-to-severe exacerbation history, and CAT score	HR 2.54 (1.01-6.36)	positive association
Gu 2021	Frailty index	mortality	154	Difference between survivors and non-survivors compared using propensity score matching (in hospital mortality) - PSM included age, sex, smoking, alcohol and comorbidities	FI higher in non-survivors compared to survivors	positive association
Scarlata 2021	Frailty index	mortality	150	CoxPH model adjusted for age and sex	HR 2.1 (0.7–5.8)	neutral association
Warwick 2021	Clinical frailty	Survival to ITU	390	Logistic regression model adjusted for	OR 4.12 (2.26-6.95)	positive association

	scale	discharge		age, sex, Multiple organ dysfunction syndrome (per 1 point), home o2, DNACRP		
COPD Exacerbations						
Gale 2018	Frailty index	exacerbations	520	Cross sectional association between FI and exacerbations in previous year - self reported frequency	Spearman's rank correlation between FI and number of exacerbations 0.327 (p<0.001)	positive association
Yee 2020	Frailty phenotype (adapted)	exacerbations	280	Association not significant in adjusted models (exacerbations treated with prednisone and/or antibiotics, treated in the emergency department or requiring hospitalization.)	IRR 1.14 (0.69-1.89)	neutral association
Ierodiakonou 2019	FiND	exacerbations	257	Cross sectional association between frailty and exacerbations in previous year - logistic regression dichotomising exacerbations (0-1 or >1 in the past year)	Higher exacerbation frequency associated with greater odds of frailty OR 1.73 (1.07-2.78)	positive association
Gephine 2021	Frailty phenotype	exacerbations	55	Chi squared test comparing proportion with 2 or more exacerbations in the past year between frailty and robust groups	No significant difference (64% vs 68%, p=0.77)	neutral association
Luo 2021	Frailty phenotype	exacerbations	309	Poisson regression model comparing exacerbation rate in frail versus non-frail (robust and pre-frail pooled) adjusted for age, gender, CCI, medication, GOLD severity, moderate-to-severe exacerbation history, and CAT.	IRR 1.75 (1.09-2.82)	positive association
Bernabeu-Mora 2020	Frailty phenotype	exacerbations		Unadjusted odds ratio for the association between exacerbation frequency (>1 per year = frequent) and the outcome categorical outcome of worsening or improving frailty status	OR 0.1 (0.01-0.58) indicating frequent exacerbations associated with worsening frailty status.	positive association
Chen 2018	Clinical frailty scale	exacerbations	125	Exacerbation frequency (2 or more per year) was associated with frailty status in an unadjusted (univariate) logistic regression model (no adjusted analysis performed on whole sample)	OR 1.89 (1.03-3.46)	positive association
Dias 2020	FRAIL	exacerbations	153	Chi squared test for association between frailty status and a dichotomised variable for exacerbation frequency (0-1 per year vs 2 or more)	No significant association (p=0.88)	neutral association

Maddocks 2016	Frailty phenotype	exacerbations	816	Cross sectional association between frailty status and exacerbation frequency in the past year	2.1 (sd 2.3) vs 3.1 (sd 3.0), p<0.001	positive association
Medina-Mirapeix 2018	Frailty phenotype	exacerbations	137	Chi squared test for association between frailty status and a dichotomised variable for exacerbation frequency (0-1 per year vs 2 or more)	No significant association (p=0.52)	neutral association
Scarlata 2021	Frailty index	exacerbations	150	Comparison of mean frailty index in people with and without frequent exacerbations (2 or more per year)	Exacerbation frequency associated with higher FI (0.15 vs 0.18, p=0.014)	positive association
Hospitalisation						
Kennedy 2019	Frailty phenotype (adapted)	hospitalisation	799 (257)	CoxPH model assessing time to first hospitalisation adjusted for components of the ADO index (age, modified Medical Research Council [mMRC] scale, and baseline forced expiratory volume in 1 second [FEV1]) sex, and lung volume reduction surgery	HR 1.8 (1.1-2.9)	positive association
Yee 2020	Frailty phenotype (adapted)	hospitalisation	280	Association not significant in adjusted models (exacerbations treated with prednisone and/or antibiotics, treated in the emergency department or requiring hospitalization.)	IRR 1.96 (0.92-4.19)	neutral association
Luo 2021	Frailty phenotype	hospitalisation	309	Incidence rate ratio for hospitalisation in frail compared to non-frail participants adjusted for age, gender, CCI, medication, GOLD severity, moderate-to-severe exacerbation history, and CAT	IRR 1.39 (1.03-1.87)	positive association
Bernabeu-Mora 2017	Edmonton	Hospitalisation – 90 day readmission	107	Logistic regression assessing assocaition between frailty and 30-day readmission following COPD exacerbation (adjusted for age, number of hospitalizations because of exacerbations in the previous year, length of stay, comorbidities and dyspnoea score)	OR for severe frailty 5.19 (1.26–21.50)	positive association
Airflow limitation						
Bernabeu-Mora 2017	Edmonton	FEV1	107	Descriptive statistics of mean FEV1 (% predicted) in robust, mild, moderate and severe frailty groups.	No signficant difference in mean %predicted FEV1 between robust (54.5), mild	neutral association

					(48.9), moderate (48.0) and severe frailty (54.2) groups. P=0.295	
Gale 2018	FI	FEV1	520	Correlation between FI and FEV1 (%) score	Higher FI correlated with lower % predicted FEV1 (-0.189, p<0.001)	positive association
Lahousse 2016	Frailty phenotype	FEV1	402	Regression model assessing the odds of frailty based on severity of airflow limitation adjusted for age, sex, pack-years of smoking, and comorbidity count.	"When classified according to severity of airflow limitation, participants with severe-COPD had a 10-fold increased risk of frailty compared with participants with a normal lung function (OR 10.0, 95% CI: 3.84–26.30, p < .001). Participants with mild-COPD were not significantly more frequently frail. However, they were more frequently prefrail, defined as having one or two frailty characteristics (OR 1.4, 95% CI: 1.01–1.89, p = .046, adjusted for age and sex; OR 1.4, 95% CI: 0.99–1.87, p = .061)."	positive association
Yee 2020	Frailty phenotype (adapted)	FEV1	280	Mean FEV1 (% predicted) compared between robust, pre-frail and frail groups.	Lower % predicted FEV1 in frail (36.9) compared with pre-frail (46.9) and robust (49.5) participants. P<0.001	positive association
Dias 2020	FRAIL scale	FEV1	203	Cross sectional analysis (unadjusted) of association between frailty status and FEV1	% predicted FEV1 lower in frail (mean 44%) compared to prefrail (52%) and robust (62%) groups, p=0.01	positive association
Hirai 2019	Kihon	FEV1	201	Correlation coefficients assessing correlation between each frailty model and FEV1	Significant correlation between frailty and lower FEV1 for each frailty model (−0.2452for Kihon, p<0.001, −0.2823 for revised frailty phenotype, p<0.001, −0.2102 for SOF, p<0.001)	positive association
Hirai 2019	Frailty phenotype (adapted)					positive association
Hirai 2019	SOF					positive association

Medina-Mirapeix 2018	Frailty phenotype	FEV1	137	Cross sectional analysis (unadjusted) of association between frailty status and FEV1	Mean % predicted FEV1 43% in frail group, 51% in prefrail group and 50% in robust group, p=0.269	neutral association
Gephine 2021	Frailty phenotype	FEV1	55	Comparison of mean % predicted FEV1 between frail and 'non-frail' (pre-frail or robust) groups.	No significant difference between frail (30) and non-frail (36) participants. P=0.18	neutral association
Luo 2021	Frailty phenotype	FEV1	309	Comparison between % predicted FEV1 of frail and 'non-frail' groups	Median FEV1 71% predicted in frail group compared to 76% in non-frail group, p<0.028	positive association
Park 2021	Tilburg	FEV1	417	Comparison of mean % predicted FEV1 between frail and non-frail participants. Unadjusted odds ratio for frailty per 1-point increase in % predicted FEV1.	Lower FEV in frail (76.7) compared with non-frail (80.1) participants. OR for frailty lower per increase in FEV1 (0.98, 95%CI 0.96-0.99)	positive association
Scarlata 2021	Frailty index	FEV1	150	Cross sectional analysis correlation between frailty index and FEV1	Significant correlation between higher FI and lower % predicted FEV1 (-0.29, p<0.01)	positive association
Dyspnoea						
Bernabeu-Mora 2017	Edmonton	dyspnoea	107	Cross-sectional comparison of MRC score severity (0-2, 3 and 4) and frailty (categorised robust, mild, moderate and severe)	Significant association between frailty and dyspnoea, e.g. percentage with MRC 4 was 19.6% in robust, 42.1% in mild, 56.3% in moderate and 84.2% in severe frailty.	positive association
Gale 2018	FI	dyspnoea	520	Correlation between FI and MRC score	Higher FI correlated with higher MRC dyspnoea score (0.466, p<0.001)	positive association
Galizia 2011	Frailty staging system	dyspnoea	489	Analysis of cross sectional association with 'effort dyspnoea'	Dyspnoea reported more commonly in moderate (51.7%) or severe (51.2%) frailty compared to robust (20.4%) or mildly frail (21.5%) participants.	positive association
Dias 2020	FRAIL scale	dyspnoea	203	Analysis of cross sectional association with baseline MRC score	Significant association between frailty and dyspnoea (mean MRC score	positive association

					4 in frail group, 2.5 in pre-frail and 2.0 in robust group, p<0.001)	
Chen 2018	Clinical frailty scale	dyspnoea	125	COPD sample divided into dyspnoea and non-dyspnoea groups. Frailty prevalence in each group assessed.	26% frailty prevalence in the non-dyspnoea group compared to 85.9% in the dyspnoea group	positive association
Hirai 2019	Kihon	dyspnoea	201	Correlation coefficients assessing correlation between each frailty model and MRC score	Significant correlation between frailty and higher MRC scored for each frailty model (0.589 for Kihon, p<0.001, 0.4753 for revised frailty phenotype, p<0.001, 0.3464 for SOF, p<0.001)	positive association
Hirai 2019	Frailty phenotype (adapted)					positive association
Hirai 2019	SOF					positive association
Ierodiakonou 2019	FiND	dyspnoea	257	Chi squared test comparing dichotomised MRC score (0-1 and >=2) and frailty categories.	Frail group had 68% in high CAT group compared to 27% in non-frail group. P<0.001	positive association
Kusunose 2017	Kihon	dyspnoea	79	Correlation coefficients assessing correlation between frailty and BDI (baseline dyspnoea index)	Significant correlation between frailty and BDI score (-0.46, p<0.01)	positive association
Medina-Mirapeix 2018	Frailty phenotype	dyspnoea	137	Prevalence of dyspnoea (MRC score 2 or more) in robust, pre-frail or frail participants.	Dyspnoea more prevalent among frail (83.3%) compared to pre-frail (34.7%) and robust (16.7%) participants.	positive association
Oishi 2020	Kihon	dyspnoea	128	Reports frailty prevalence at different levels of MRC score	MRC 0 – 13.9% frail<br>MRC 1 – 8.3% frail<br>MRC 2 – 43.3% frail<br>MRC 3 – 52.6% frail<br>MRC 4 – 94.7% frail	positive association
Park 2013	Frailty index	dyspnoea	211	Odds ratio for frailty (dichotomised) assocaited with shortness of breath on stairs or inclines	OR for frailty 3.94 (2.03-7.62) - unadjusted	positive association
Gephine 2021	Frailty phenotype	dyspnoea	55	Mean MRC score compared between frail and non-frail (robust or pre-frail) groups	No significant difference between groups (3.4 vs 3.0, p=0.07)	neutral association
Luo 2021	Frailty phenotype	dyspnoea	309	Comparison between MRC score of frail and 'non-frail' groups	Median CAT score 2 in frail group compared to 1 in non-frail group, p<0.001. 49% of frail group had MRC >1, 6%	positive association

					of non-frail group, p<0.001	
Scarlata 2021	Frailty index	dyspnoea	150	Cross sectional analysis of the correlation between FI and MRC score.	Higher FI correlated with higher MRC score (0.512, p<0.01)	positive association
Severity measures						
Dias 2020	FRAIL scale	severity	203	Mean CAT score reported for robust, pre-frail and frail groups	Higher mean CAT score in frail (20) versus pre-frail (13) and robust (5) groups. P<0.001	positive association
Hirai 2019	Kihon	severity	201	Correlation coefficients assessing correlation between each frailty model and CAT score	Significant correlation between frailty and higher CAT scored for each frailty model (0.6014 for Kihon, p<0.001, 0.4051 for revised frailty phenotype, p<0.001, 0.3492 for SOF, p<0.001)	positive association
Hirai 2019	Frailty phenotype (adapted)					positive association
Hirai 2019	SOF					positive association
Ierodiakonou 2019	FiND	severity	257	Chi squared test comparing dichotomised CAT score (<=10 and >10) and frailty categories.	Frail group had 93% in high CAT group compared to 77% in non-frail group. P=0.002	positive association
Kusunose 2017	Kihon	severity	79	Correlation coefficients assessing correlation between frailty and CAT score	Significant correlation between frailty and higher CAT score (0.38, p<0.01)	positive association
Medina-Mirapeix 2018	Frailty phenotype	severity	137	Comparison between mean CAT score in robust, pre-frail and frail participants.	Higher mean CAT score in frail (18.4) compared to prefrail (14.4) and robust (11.4) participants. P=0.21	positive association
Scarlata 2021	Frailty index	severity	150	CAT score compared between dichotomised FI (low vs high) groups.	Higher CAT scores in high FI group (14.8 vs 11.1, p=0.01)	positive association
Luo 2021	Frailty phenotype	severity	309	Comparison between CAT score of frail and 'non-frail' groups	Median CAT score 12 in frail group compared to 5 in non-frail group, p<0.001	positive association
Gale 2018	Frailty index	severity	520	Correlation between FI and CAT score	Higher FI correlated with higher CAT score (0.594, p<0.001)	positive association
Disability						
Medina-Mirapeix 2016	Edmonton	disability	103	Association between frailty status before admission and functional decline after hospitalisation with COPD exacerbation.	Frailty associated with functional decline after COPD exacerbation (OR 3.97; 95% CI 1.13–13.92)	positive association

Quality of life						
Kennedy 2019	Frailty phenotype (adapted)	quality of life	902	Comparison of SF-36 quality of life scores compared frail participants with prefrail or robust participants.	"frail patients reported consistently lower scores (indicating worse functioning) than the other patients for SF-36 physical functioning (mean difference 216.7; 95% CI, 221.3 to 212.1; P , 0.0001) and physical composite (mean difference 25.5; 95% CI, 27.6 to 23.4; P = 0.0001) scores"	positive association
Yee 2020	Frailty phenotype (adapted)	quality of life	280	SF-36 score compared between robust, pre-frail and frail groups	Lower overall mean SF-36 scores in frail (39.2) compared to pre-frail (46.2) and robust (60.9) participants.	positive association
Kusunose 2017	Kihon	quality of life	79	Correlation coefficients assessing correlation between frailty and SGRQ score	Significant correlation between frailty and higher SGRQ score (0.65, p<0.01)	positive association
Gephine 2021	Frailty phenotype (adapted)	quality of life	55	Comparison of Clinical COPD Questionnaire scored between frailty and non-frail groups	Lower mean CCQ scores in frail (2.6) compared to non-frail (3.4) participants (p=0.01)	neutral association

Funnel plots

Supplementary figure 3 - Mortality meta-analysis funnel plot

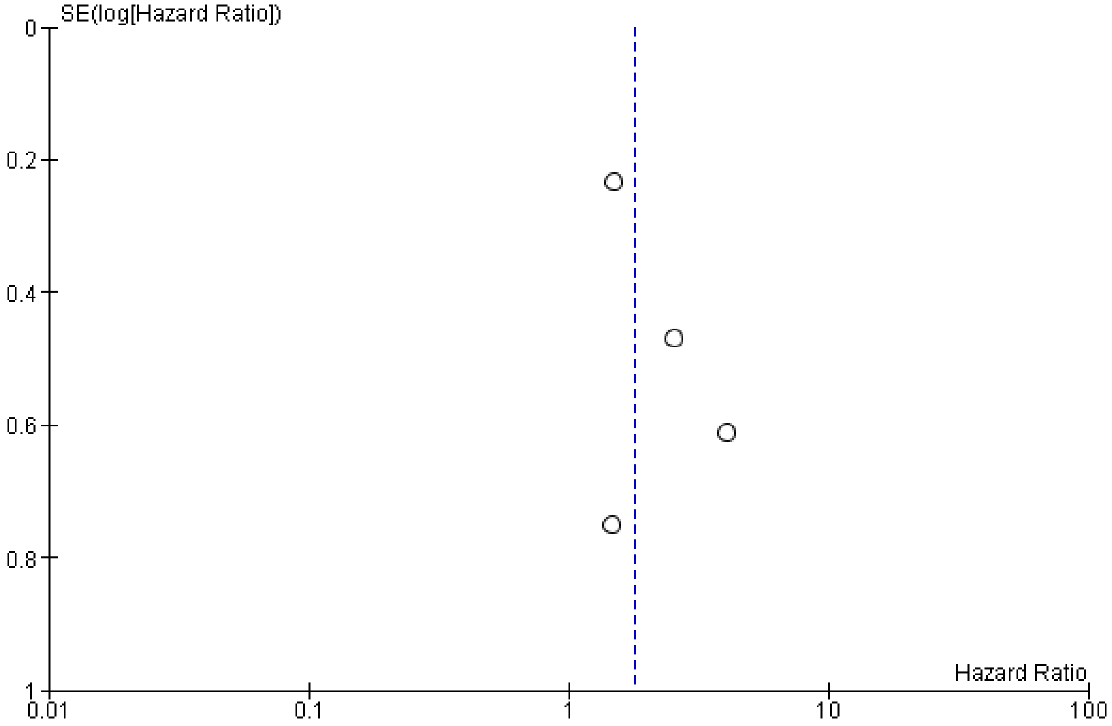

Supplementary figure 4 - Hospitalisation meta-analysis funnel plot

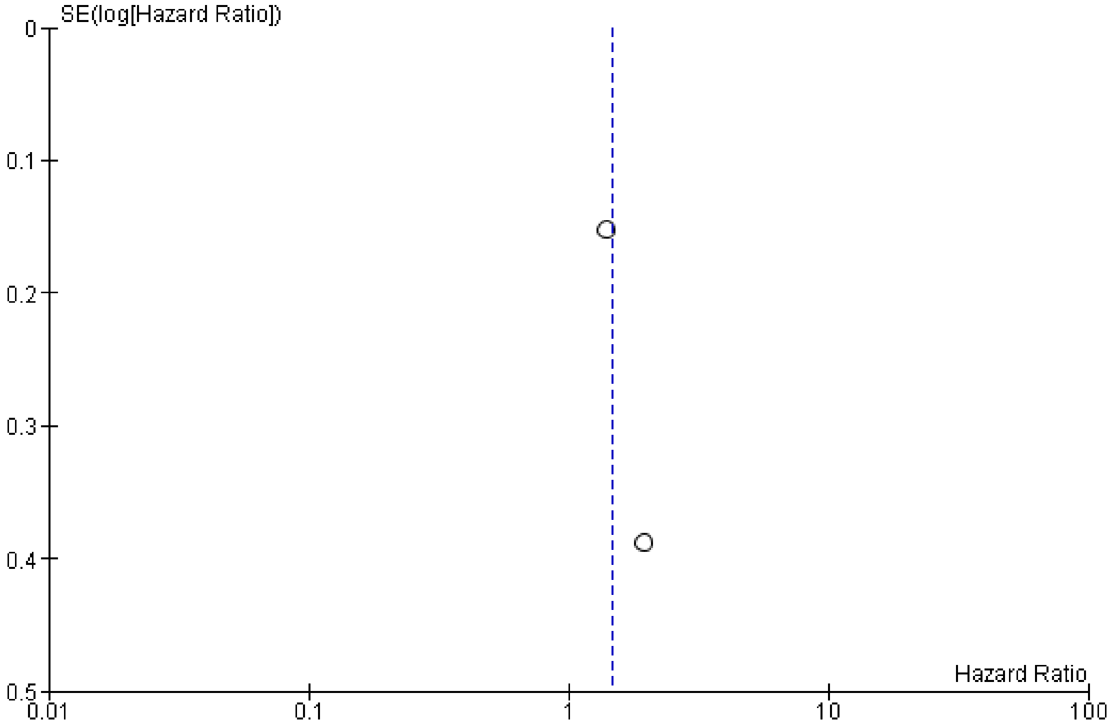

Supplementary figure 5 - COPD exacerbation meta-analysis funnel plot

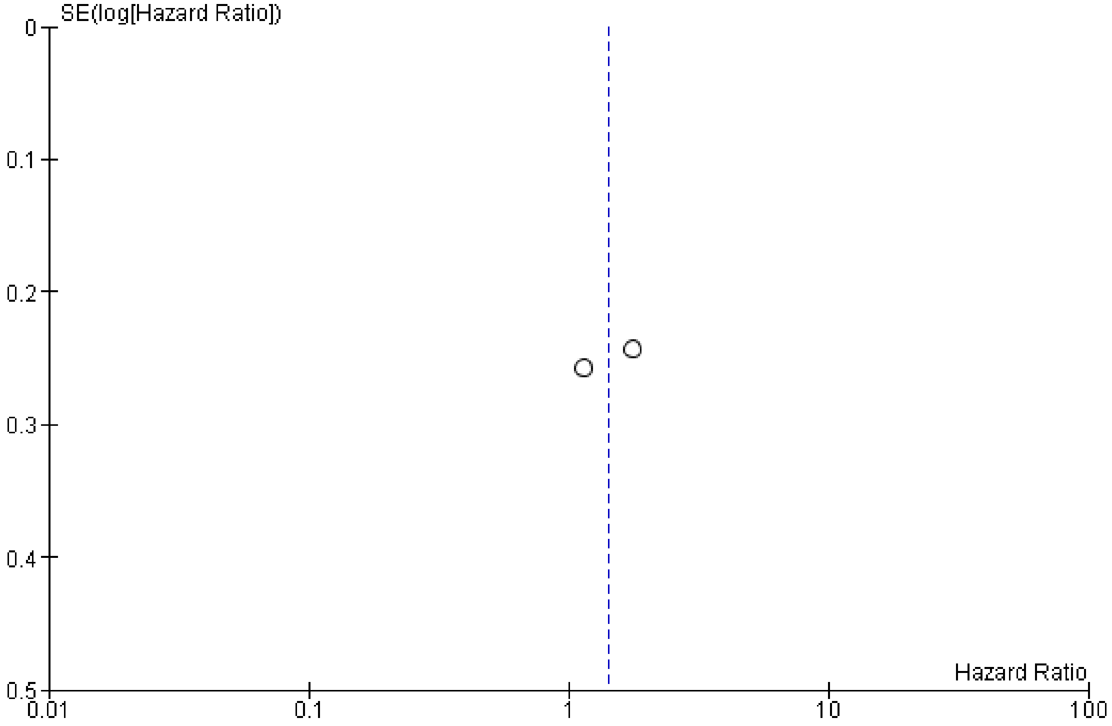

Supplementary table 2 - Quality assessment individual study assessments

Author	Year	01_representative	01_comment	02_non_exposed	02_comment	03_exposure	03_comment	04_non_respondents	04_comment	05_outcome_not_present_at_start	05_comment	06_cofounders	06_comment	07_outcome_assessment	07_comment	08_followup_length	08_comment	09_followup_completeness	09_comment
Akgun, K. M.-//-Tate, J. P.-//-Oursler, K. K.-//-Crystal, S.-//-Leaf, D. A.-//-Womack, J. A.-//-Brown, T. T.-//-Justice, A. C.-//-Crothers, K.	2016	0	not representative sample - matched to HIV infected population and VES cohort not nationally representative	1		1	adaptation of frailty phenotype but fully described in text and references	0											
Ambagtsheer, R. C.-//-Beilby, J.-//-Seiboth, C.-//-Dent, E.	2019	1	specifically recruited care-homes so may not be generalisable - however no selection bias within carehome residents - somewhat representative (or target population)	1		1		0											
Avila-Funes, J. A.-//-Helmer, C.-//-Amieva, H.-//-Barberger-Gateau, P.-//-Le Goff, M.-//-Ritchie, K.-//-Portet, F.-//-Carriere, I.-//-Tavernier, B.-//-Gutierrez-Robledo, L. M.-//-Dartigues, J. F.	2008	1	Somewhat representative - excludes institutionalised individuals which may limit representativeness and selectively exclude people with frailty	1		2		0											
Bernabeu-Mora, R.-//-Garcia-Guillamon, G.-//-Valera-Novella, E.-//-Gimenez-Gimenez, L. M.-//-Escolar-Reina, P.-//-Medina-Mirapeix, F.	2017	1	Somewhat representative - excludes cognitive impairment (MMSE <20)	1		1	Edmonton frailty scale recorded by research assistant for all participants	1	Some excluded due to longer length of stay - acceptable given focus on hospital readmission	1	outcome readmission	2	Final Model included frailty, age, number of hospitalizations because of exacerbations in the previous year, length of stay, comorbidities and dyspnoea score	1		1	90-day readmission was outcome - all participants followed for this timeframe	1	
Bernabeu-Mora, R.-//-Oliveira-SoUnited States, S. L.-//-Sanchez-Martinez, M. P.-//-Garcia-Vidal, J. A.-//-Gacto-Sanchez, M.-//-Medina-Mirapeix, F.	2020	1	Somewhat representative - exclusion of 'cognitive deterioration' - outpatient sample so may reflect more severe disease - but prospectively sampled from all clinic attendees.	1		2		0		1	Frailty trajectories assessed - so comparable assessment of frailty beginning and end	2		1		1	2-year followup with demonstrable change in frailty status within study population over this time.	1	18/137 dropped out with reasons accounted for.
Blaum, C. S.-//-Xue, Q. L.-//-Michelon, E.-//-Semba, R. D.-//-Fried, L. P.	2005	1	Somewhat representative - women only but repreentative sampling of women within this population	1		2		0											
Castellana, Fabio-//-Lampignano, Luisa-//-Bortone, Ilaria-//-Zupo, Roberta-//-Lozupone, Madia-//-Griseta, Chiara-//-Daniele, Antonio-//-De Pergola, Giovanni-//-Giannelli, Gianluigi-//-Sardone, Rodolfo	2021	1	somewhat representative - registry used to obtain representative initial sample, analysis then performed on subsample that completed all assessments, the representativeness of which is not clear	1		2		0	Excluded if not completing 'all assessments' but details of these individuals not compared to the full sample										
Chen, Chin-Ying-//-Wu, Shwu-Chong-//-Chen, Liang-Ju-//-Lue, Bee-Horng	2010	1		1		2		0											
Chen, P. J.-//-Yang, K. Y.-//-Perng, W. C.-//-Lin, K. C.-//-Wang, K. Y.	2018	0	inclusion criteria not fully described	1		2		0	no details										
Cheong, C. Y.-//-Nyunt, M. S. Z.-//-Gao, Q.-//-Gwee, X.-//-Choo, R. W. M.-//-Yap, K. B.-//-Wee, S. L.-//-Ng, T. P.	2019	1		1		2		0											
Chin, M.-//-Voduc, N.-//-Huang, S.-//-Forster, A.-//-Mulpuru, S.	2020	0	specific respiratory ward in tertiary centre	1		2		0											
Crow, R. S.-//-Lohman, M. C.-//-Titus, A. J.-//-Bruce, M. L.-//-Mackenzie, T. A.-//-Bartels, S. J.-//-Batsis, J. A.	2018	1	NHANES survey	1		1	adaptation of frailty phenotype	0											
de Albuquerque SoUnited States, Ana Carolina Patrício-//-Dias, Rosângela Correa-//-Maciel, Álvaro Campos Cavalcanti-//-Guerra, Ricardo Oliveira	2012	0	specific to a region within one area of Brazil - recognised as a socioeconomically deprived region so likely to influence	1		2		0											

			prevalence - also excluded various conditions including cognitive impairment, parkinson's, stroke																
Dias, L. D.-//-Ferreira, A. C. G.-//-da Silva, J. L. R.-//-Conte, M. B.-//-Rabahi, M. F.	2020	0	single clinic proving free treatment - potential for selection bias	1		2		0											
Fragoso, Carlos A Vaz-//-Enright, Paul L-//-McAvay, Gail-//-Van Ness, Peter H-//-Gill, Thomas M	2012	1	somewhat representative - excludes some with conditions likely to impact frailty status (e.g. cognitive impairment, stroke)	1		2		0											
Fried, Linda P.-//-Tangen, Catherine M.-//-Walston, Jeremy-//-Newman, Anne B.-//-Hirsch, Calvin-//-Gottdiener, John-//-Seeman, Teresa-//-Tracy, Russell-//-Kop, Willem J.-//-Burke, Gregory	2001	1	somewhat representative - excludes some with conditions likely to impact frailty status (e.g. cognitive impairment, stroke)	1		2		0											
Gale, N. S.-//-Albarrati, A. M.-//-Munnery, M. M.-//-Hubbard, R. E.-//-Tal-Singer, R.-//-Cockcroft, J. R.-//-Shale, D. J.	2018	1	somewhat representative - few exclusions but designed to reflect 'general COPD population'	1		2		0											
Galizia, G.-//-Cacciatore, F.-//-Testa, G.-//-Della-Morte, D.-//-Mazzella, F.-//-Langellotto, A.-//-Raucci, C.-//-Gargiulo, G.-//-Ferrara, N.-//-Rengo, F.-//-Abete, P.	2011	1	Representative - random sample from electoral role	1		1		0	Details of non-respondents not reported other than 35% refusal rate - potential for bias	1	mortality assessed by death certificate	2	age, sex, BMI, heart rate, pulse pressure, effort dyspnea, Charlson comorbidity index, drug number, β-agonists, steroids, current smoking, former smoking, MMSE, GDS, BADL (≥1 lost) and social support	1		1	12 years	1	35 not reachable out of total sample 1332
Gephine, S.-//-Mucci, P.-//-Grosbois, J. M.-//-Maltais, F.-//-Saey, D.	2021	1	sampling of those referred for pulmonary rehabilitation	1		2		1											
Gu, J. J.-//-Liu, Q.-//-Zheng, L. J.	2021	1		1		1	FI-lab created for the study itself but according to well-described criteria	1											
Hanlon, P.-//-Nicholl, B. I.-//-Jani, B. D.-//-Lee, D.-//-McQueenie, R.-//-Mair, F. S.	2018	0	UK Biobank non-representative sample	1		1	adaptation of frailty phenotype	0	low response rate										
Hirai, K.-//-Tanaka, A.-//-Homma, T.-//-Kaneko, K.-//-Uno, T.-//-Sato, H.-//-Manabe, R.-//-Ohta, S.-//-Kusumoto, S.-//-Yamaguchi, F.-//-SuzUnited Kingdomi, S.-//-Sagara, H.	2019	1	consencutive recruitment within outpatients - single centre	1		1		0											
Ierodiakonou, D.-//-Kampouraki, M.-//-Poulonirakis, I.-//-Papadokostakis, P.-//-Lintovoi, E.-//-Karanassos, D.-//-Maltezis, K.-//-Chorti, M.-//-Petrovitsos, E.-//-Dimopoulou, S.-//-Hamind, S.-//-Gialamas, I.-//-Athanasiou, P.-//-Bempi, V.-//-Lambraki, I.-//-Tsiligianni, I.	2019	0	selective sampling	1		1		0											
Kennedy, C. C.-//-Novotny, P. J.-//-LeBrasseur, N. K.-//-Wise, R. A.-//-Sciurba, F. C.-//-Benzo, R. P.-//-Fishman, A. P.-//-Bozzarello, B. A.-//-Al-Amin, A.-//-Katz, M.-//-Wheeler, C.-//-Baker, E.-//-Barnard, P.-//-Carter, J.-//-Chatziioannou, S.-//-Conejo-Gonzales, K.-//-Haddad, J.-//-Hicks, D.-//-, C.-//-Livingston, H.-//-Meinert, J.-//-Meyers, J	2019	1		1		1	adaptation of frailty phenotype	0		1		2		1		1		1	
Kim, S.-//-Jung, H. W.-//-Won, C. W.	2020	0	not representative sample - selective recruitment and exclusion crtieria	1		1	adaptation of frailty phenotype	0											
Kusunose, M.-//-Oga, T.-//-Nakamura, S.-//-Hasegawa, Y.-//-Nishimura, K.	2017	1	consencutive recruitment within outpatients - single centre	1		1		0											
Lahousse, L.-//-Maes, B.-//-Ziere, G.-//-Loth, D. W.-//-Verlinden, V. J. A.-//-Zillikens, M. C.-//-Uitterlinden, A. G.-//-Rivadeneira, F.-//-Tiemeier, H.-//-Franco, O. H.-//-Ikram, M. A.-//-Hofman, A.-//-Brusselle, G. G.-//-Stricker, B. H.	2014	1	Rotterdam study assessed to be representative of Netherlands population over 55	1		1	adaptation of frailty phenotype	0											
Lahousse, L.-//-Ziere, G.-//-Verlinden, V. J. A.-//-Zillikens, M. C.-//-Uitterlinden, A. G.-//-Rivadeneira, F.-//-Tiemeier, H.-//-Joos, G. F.-//-Hofman, A.-//-Ikram, M. A.-//-Franco, O. H.-//-Brusselle, G. G.-//-Stricker, B. H.	2016	1	Rotterdam study assessed to be representative of Netherlands population over 55	1		1	adaptation of frailty phenotype	0		1		2		1		1		1	
Lai, H. Y.-//-Chang, H. T.-//-Lee, Y. L.-//-Hwang, S. J.	2014	0	Male only veterens care home - findings likely specific to this setting	1		1		0											

Lee, Jenny S. W.-//-Auyeung, Tung-Wai-//-Leung, Jason-//-Kwok, Timothy-//-Woo, Jean	20 14	1		1		1		0											
Limpawattana, P.-//-Putraveephong, S.-//-Inthasuwan, P.-//-Boonsawat, W.-//-Theerakulpisut, D.-//-Chindaprasirt, J.	20 17	0	various exclusions based on functional impairment - likely to influence frailty prevalence	1		2		0											
Liotta, Giuseppe-//-O'Caoimh, Rónán-//-Gilardi, Francesco-//-Proietti, Maria Grazia-//-Rocco, Gennaro-//-Alvaro, Rosaria-//-Scarcella, Paola-//-Molloy, D William-//-Orlando, Stefano-//-Mancinelli, Sandro	20 17	1	random sampling - but regionally specific	1		0	little descripti on of critieria	0											
Luo, J.-//-Zhang, D.-//-Tang, W.-//-Dou, L. Y.-//-Sun, Y.	20 21	1	somewhat representative - consecutive recruitment but based in single teriary centre	1		2		0		1		2		1		1		1	
Ma, L.-//-Tang, Z.-//-Zhang, L.-//-Sun, F.-//-Li, Y.-//-Chan, P.	20 18	1	stratified sampling from 7 cities	1		2		0											
Maddocks, M.-//-Kon, S. S. C.-//-Canavan, J. L.-//-Jones, S. E.-//-Nolan, C. M.-//-Labey, A.-//-Polkey, M. I.-//-Man, W. D. C.	20 16	1		1		2		0		1		2		1		1		1	
Medina-Mirapeix, F.-//-Bernabeu-Mora, R.-//-Garcia-Guillamon, G.-//-Valera Novella, E.-//-Gacto-Sanchez, M.-//-Garcia-Vidal, J. A.	20 16	0	exclusions criteria likely to be restictive for people with frailty (cognitive, excluded if not reviewed for COPD in prev 6 months, longer length of stay)	1		2		0											
Medina-Mirapeix, F.-//-Bernabeu-Mora, R.-//-Gimenez-Gimenez, L. M.-//-Escolar-Reina, P.-//-Gacto-Sanchez, M.-//-de Oliveira-SoUnited States, S. L.	20 18	1	somewhat - consecutive recruitment in outpatients but based on a single centre	1		2		0											
Motokawa, K.-//-Watanabe, Y.-//-Edahiro, A.-//-Shirobe, M.-//-Murakami, M.-//-Kera, T.-//-Kawai, H.-//-Obuchi, S.-//-Fujiwara, Y.-//-Ihara, K.-//-Tanaka, Y.-//-Hirano, H.	20 18	1		1		2		0											
Nagorni-Obradovic, L. M.-//-VUnited Kingdomovic, D. S.	20 14	0		1		1		0											
Oishi, K.-//-Matsunaga, K.-//-Harada, M.-//-Suizu, J.-//-Murakawa, K.-//-Chikumoto, A.-//-Ohteru, Y.-//-Matsuda, K.-//-Uehara, S.-//-Hamada, K.-//-Ohata, S.-//-Murata, Y.-//-Yamaji, Y.-//-Asami-Noyama, M.-//-Edakuni, N.-//-Kakugawa, T.-//-Hirano, T.	20 20	1	somewhat - consecutive recruitment in outpatients but based on a single centre	1		2		0											
Park, S. K.-//-Richardson, C. R.-//-Holleman, R. G.-//-Larson, J. L.	20 13	1	NHANES survey	1		0	only some of Tiburg criteria available within dataset	0											
Park, S. K.	20 21	1	KNHANES - nationally representative	1		0	only some of Tiburg criteria available within dataset	0											
Pollack, Lauren R.-//-Litwack-Harrison, Stephanie-//-Cawthon, Peggy M.-//-Ensrud, Kristine-//-Lane, Nancy E.-//-Barrett-Connor, Elizabeth-//-Dam, Thuy-Tien	20 17	1		1		1		0											
Scarlata, S.-//-Finamore, P.-//-Laudisio, A.-//-Cardaci, V.-//-Ramaccia, M.-//-D'Alessandro, F.-//-Pedone, C.-//-Antonelli Incalzi, R.-//-Cesari, M.	20 21	0	outpatient recruitment with exclusion of various comorbidities	1		2		0		1		1		1		1		1	
Serra-Prat, M-//-Papiol, M-//-Vico, J-//-Palomera, E-//-Sist, X-//-Cabré, M	20 16	1	somewhat - selected from primary care centres with good population coverage. Some exlusions present regarding cognition and institutionalisation	1		1	adapted frailty phenoty pe	0											
ter Beek, L.-//-van der Vaart, H.-//-Wempe, J. B.-//-Krijnen, W. P.-//-Roodenburg, J. L. N.-//-van der Schans, C. P.-//-Jager-Wittenaar, H.	20 20	0	somewhat - exclusions included wheelchair use, skin problems, cognitive impairment. Likey to exclude more severe frailty	1		1		0											
Uchmanowicz, I.-//-Jankowska-Polanska, B.-//-Chabowski, M.-//-Uchmanowicz, B.-//-Fal, A. M.	20 16	0	insufficient detail to assess	1		1		0											
Valenza, M. C.-//-Torres-Sanchez, I.-//-Cabrera-Martos, I.-//-Rodriguez-Torres, J.-//-Gonzalez-Jimenez, E.-//-Munoz-Casaubon, T.	20 16	0	multiple exclusion crtieria based on comorbidities, method of identification not clear	1		1		0											
Veronese, Nicola-//-Sigeirsdottir, Kristin-//-Eiriksdottir, Gudny-//-Marques, Elisa A.-//-Chalhoub, Didier-//-Phillips, Caroline L.-//-Launer, Lenore J.-//-Maggi, Stefania-//-	20 17	1		1		1		0											

Gudnason, Vilmundur-//-Harris, Tamara B.																			
Warwick, M.-//-Fernando, S. M.-//-Aaron, S. D.-//-Rochwerg, B.-//-Tran, A.-//-Thavorn, K.-//-Mulpuru, S.-//-McIsaac, D. I.-//-Thompson, L. H.-//-Tanuseputro, P.-//-Kyeremanteng, K.-//-Resource Optimization, Network	20 21	1	representative of ITU (target) population, although no generalisable beyond this	1		1		0		1		1		1		1		1	
Xue, Q.-//-Qin, M. Z.-//-Jia, J.-//-Liu, J. P.-//-Wang, Y.	20 19	0		1		1		0											
Yee, N.-//-Locke, E. R.-//-Pike, K. C.-//-Chen, Z. J.-//-Lee, J.-//-Huang, J. C.-//-Nguyen, H. Q.-//-Fan, V. S.	20 20	1	Rotterdam study assessed to be representative of Netherlands population over 55	1		1	adaptati on of frailty phenoty pe	0	no details	1	prospectiv e assessme nt of mortality, hospitalis ation, exacerbati ons	1	adjusted for age, sex, Charlson comorbidity index ≥1, supplemental home oxygen use, COPD hospitalizations in the prior year, FEV1%predicted, and mMRC score	1		1		1	
Zhang, Y.-//-Li, Y.-//-Chan, P.-//-Ma, L.	20 20	0		1		1		0											
